# The Emerging Role of Microbiota and Microbiome in Pancreatic Ductal Adenocarcinoma

**DOI:** 10.3390/biomedicines8120565

**Published:** 2020-12-03

**Authors:** Sona Ciernikova, Maria Novisedlakova, Danka Cholujova, Viola Stevurkova, Michal Mego

**Affiliations:** 1 Department of Genetics, Cancer Research Institute, Biomedical Research Center of the Slovak Academy of Sciences, 845 05 Bratislava, Slovakia; viola.stevurkova@savba.sk; 2 Oncology Outpatient Clinic, Hospital of the Hospitaller Order of Saint John of God, 814 65 Bratislava, Slovakia; maria.novisedlakova@gmail.com; 3 Department of Tumor Immunology, Cancer Research Institute, Biomedical Research Center of the Slovak Academy of Sciences, 845 05 Bratislava, Slovakia; dana.cholujova@savba.sk; 4 2nd Department of Oncology, Faculty of Medicine, Bratislava and National Cancer Institute, Comenius University, 833 10 Bratislava, Slovakia; misomego@gmail.com

**Keywords:** pancreatic ductal adenocarcinoma (PDAC), pancreatic microbiome, immune suppression, tumor microenvironment, cancer treatment, microbiota modulation

## Abstract

Pancreatic ductal adenocarcinoma (PDAC) is one of the most aggressive malignant tumors due to the absence of biomarkers for early-stage detection and poor response to therapy. Since mounting evidence supports the role of microbiota composition in tumorigenesis and cancer treatment, the link between microbiome and PDAC has been described. In this review, we summarize the current knowledge regarding the impact of the gut and oral microbiome on the risk of PDAC development. Microenvironment-driven therapy and immune system interactions are also discussed. More importantly, we provide an overview of the clinical trials evaluating the microbiota role in the risk, prognosis, and treatment of patients suffering from PDAC and solid tumors. According to the research findings, immune tolerance might result from the microbiota-derived remodeling of pancreatic tumor microenvironment. Thus, microbiome profiling and targeting represent the potential trend to enhance antitumor immunity and improve the efficacy of PDAC treatment.

## 1. Introduction

Pancreatic ductal adenocarcinoma (PDAC) is the most prevalent neoplastic disease of the pancreas accounting for more than 90% of all pancreatic malignancies [1]. It is the fourth most frequent cause of cancer-related deaths worldwide and is projected to be the second cause of cancer-related deaths by 2030 [2]. Genetic susceptibility, age, gender, ethnicity, diabetes mellitus, as well as alcohol abuse, vitamin D deficiency, tobacco use, high body mass index, and lack of physical activity tend to increase the development of PDAC [3]. Additionally, pancreatic inflammation is considered a long-term risk factor, and chronic pancreatitis supposed to raise the risk for PDAC up to 20 times [4]. The poor prognosis of PDAC is a result of its complex biology [5]. The majority of PDAC patients (80–85%) are diagnosed at late tumor stages with locally advanced or metastatic disease. Only 15–20% are diagnosed in an early stage allowing them to undergo surgical resection leading to an improvement in the 5-year survival rate to a 20–25% [6,7,8]. Current standard of care for patients with PDAC focusses on chemotherapeutic regimens and pancreatic surgery. However, limited treatment options, late diagnosis in advanced tumor stages and the aggressive behavior of PDAC contribute to the high mortality of the disease [9].

Mounting evidence identifies a key role of microbiota in maintaining the homeostatic balance within a human body. Very recently, an extensive gut microbiome analysis comprising 3409 participants has uncovered almost 150 host–microbiome connections relevant to human health and disease, showing a great potential of given relationships for future research and clinical trials [10]. Growing consensus link the human microbiome with tumorigenesis and modulation of cancer treatment. Reexamination of whole-genome and whole-transcriptome sequencing data from The Cancer Genome Atlas for microbial sequences demonstrated the associations between different cancer types and specific microbiota [11]. Mouse models and also clinical studies suggest the involvement of gut microbiome in PDAC initiation and progression through immune system modulation. Maekawa et al. observed that *E. faecalis* or lipoteichoic acid might aggravate the pancreatic inflammation by stimulating inflammatory cytokines [12]. Interestingly, PDAC samples exhibit more abundant microbiome compared to controls obtained from normal pancreas.

Here, we provide the current knowledge about the PDAC microbiome and its role in modulating the response to distinct treatment modalities. Importantly, clinical trials concerning the impact of microbiota modifications in PDAC patients will be summarized. Due to the very low survival rate, comprehensive studies related to all possible factors affecting PDAC tumorigenesis and treatment should be of highest interest. However, research findings regarding this field are limited, and a better understanding of the associations between microbial changes and efficacy of therapy might bring new perspectives for microbiota-derived approach in PDAC.

## 2. Current Therapeutic Possibilities for Pancreatic Ductal Adenocarcinoma

Despite great efforts and an increasing body of evidence from research and clinical trials, the 5-year overall survival (OS) in PDAC patients has only slightly increased from 5% to 9% [13]. This small progress was mainly achieved through recent improvements in neoadjuvant and adjuvant therapeutic strategies as well as perioperative care [14]. According to clinical data, the currently applied chemotherapeutic regimens are unlikely to be able to sufficiently improve the clinical impact of patients. Hence, the discovery of additional relevant risk factors, the identification of suitable biomarkers, and a better understanding of main players affecting the efficacy of PDAC therapy represent the important directions for upcoming research.

### 2.1. Systemic Therapy for Locally Advanced and Metastatic Tumors

5-fluorouracil (5-FU) was the principal treatment option for metastatic PDAC through the 1990s, although response rates were under 20% and median survival was just 6 months [15,16]. Other agents or combinations of drugs failed to show any improvement over 5-FU monotherapy until a landmark study in 1997 by Burris et al. demonstrated the superiority of gemcitabine for advanced PDAC [17]. The single-agent gemcitabine had been a standard of care first-line treatment for advanced disease for more than two decades until the PRODIGE and MPACT clinical trials demonstrated that two combination chemotherapy regimens, FOLFIRINOX (folinic acid, fluorouracil, irinotecan, and oxaliplatin) and gemcitabine/nab-paclitaxel, respectively, achieved higher response rates and longer median OS than gemcitabine [18,19,20]. PRODIGE was a phase II–III, open-label trial that compared FOLFIRINOX to gemcitabine for patients with advanced PDAC. FOLFIRINOX increased the median OS by 4.3 months (11.1 versus 6.8 months) [18]. In MPACT phase III, open-label trial, the patients were randomized to gemcitabine/nab-paclitaxel and gemcitabine alone arms. The median OS was 8.5 months with gemcitabine/nab-paclitaxel compared to 6.7 months with gemcitabine [20]. FOLFIRINOX and gemcitabine/nab-paclitaxel are the two current standards of care first-line treatment regimens for advanced PDAC. They have also become the chemotherapy regimens of choice for neoadjuvant therapy for borderline resectable or locally advanced pancreatic tumors [19].

Unfortunately, there are limited predictive biomarkers for therapy selection in locally advanced and metastatic pancreatic cancer. Microsatellite instability (MSI) and high tumor mutational burden (TMB) are markers predicting the effectivity of immune checkpoint inhibitors (ICI) irrespective of tumor type. In pancreatic cancer, the prevalence of MSI is very low 0–1.3%, while high TMB was observed in 27.9% of pancreatic cancer [21,22]. Increasing evidence suggests a close relationship between the gut microbiome and effectivity of ICI (see below).

Homologous recombination repair deficiency (HRD) represents another emerging biomarker for PDAC treatment decision. Somatic or germline mutations in breast cancer susceptibility *BRCA1/2* or *PALB2* genes are presented in approximately 10% of pancreatic tumors [23,24,25,26]. Cancer cells with defects in genes associated with homologous recombination repair (HRR) exhibit a deficiency in the repair of DNA double-strand breaks, with subsequent clinical implications for treatment selection. Regimens that include a platinum agent should be considered as standard regimens in these tumors [27]. Germline *BRCA* or *PALB2* mutation also selects patients with potential benefit from maintenance treatment with a poly (ADP-ribose) polymerase (PARP) inhibitor after an initial period of platinum-containing chemotherapy. In the POLO trial, 154 patients with germline *BRCA*-mutated metastatic pancreatic cancer who had not progressed during at least 16 weeks of first-line platinum-based therapy were randomly assigned to placebo or maintenance olaparib alone. The median progression-free survival was significantly longer in the olaparib group compared to the placebo group (7.4 versus 3.8 months). Similarly, the objective response rate in the maintenance arm, as well as median duration of response, was significantly better in the olaparib arm [28].

### 2.2. Systemic Therapy for Resectable Tumors

For patients with resectable PDAC, the results of the ESPAC-4 trial and the PRODIGE-24 trial have established new standards of care [29]. In the ESPAC-4 trial, patients with resected PDAC were randomized to receive adjuvant gemcitabine plus capecitabine or single-agent gemcitabine. The addition of capecitabine resulted in a statistically significant improvement in the median OS (28 versus 25.5 months) [30]. In the PRODIGE-24 trial, patients with resected PDAC were randomized to receive mFOLFIRINOX or gemcitabine. Those receiving mFOLFIRINOX demonstrated the longest median OS ever reported in this population (54.4 versus 35 months) [31]. In the JASPAC-01 trial, patients of Japanese ethnicity with resected PDAC were randomized to receive either S-1 or gemcitabine in the adjuvant setting. Patients in the S-1 arm showed 5-year OS of 44% [32]. Recently, an international, randomized, open-label, phase III trial APACT aiming to study the outcomes of adjuvant nab-paclitaxel plus gemcitabine (nab-P/Gem) versus gemcitabine alone treatment in 866 surgically resected PDAC patients (NCT01964430) showed no significant benefit in disease-free survival (DFS) for nab-P/Gem versus gemcitabine alone in the adjuvant setting of PDAC treatment. However, the interim OS and sensitivity analysis of DFS by the investigator were improved for nab-P/Gem compared to Gem, suggesting the possibility of adjuvant nab-P/Gem therapy for patients not eligible for adjuvant FOLFIRINOX [33]. 

## 3. The Role of Gut Microbiome in Cancer Treatment

The gut microbiome represents the collective genetic material within microbiota residing the human intestinal tract. Host genetics partially shapes the microbiome, but a number of other factors, such as type of birth, nutrition, age, environment, lifestyle, use of antibiotics, and anticancer treatment, contribute to its changes as well. Together, all attributes result in the unique microbial composition for each individual. The gut microbiota plays an important role in enhancing intestinal homeostasis and mucosal barrier integrity, and interactions with the host immune system have been reviewed elsewhere [34]. Importantly, a growing number of preclinical and clinical studies emphasize the impact of microbial composition on the response to chemo- and immunotherapy, suggesting the therapeutic potential of gut microbiota manipulation in cancer patients.

### 3.1. Microbiome, Chemotherapy, and Antidiabetic Drugs

Oxaliplatin represents the backbone of the FOLFIRINOX regimen in PDAC. The main effect of platinum compounds is disruption and alkylation of the tumor cell DNA, which further interfere with DNA repair and lead to subsequent cell death. Oxaliplatin has an immunomodulatory effect as well, potentiating tumoricidal T-cell immunity. The study by Iida et al. showed that ATB-treated mice produced fewer reactive oxygen species (ROS) in myeloid cells, followed by decreased efficacy and impaired survival after oxaliplatin treatment. However, the efficacy of chemotherapy was restored after administration of lipopolysaccharide. A special group represented the mice with a defective Toll-like receptor (TLR) signaling pathway, demonstrating no response to oxaliplatin treatment. Agonistic TLR molecules from microbial membranes are reported to help stimulate the immune system and increase ROS production [35].

Irinotecan is another key drug in PDAC treatment, characterized by relatively common side effects such as diarrhea, limiting the dose and effectiveness of treatment. Enzymatic activity of intestinal bacteria is known to influence the course of side effects. Some bacteria are beneficial in this case, while others worsen the toxicity. Importantly, the composition of gut microbiota in cancer patients tends to be in imbalance due to the disease itself and the use of other chemotherapeutics. The active antitumor metabolite of irinotecan in the blood is SN-38, which is conjugated in the liver by glucuronidase to the inactive form of SN-38G and is excreted by the biliary tract into the intestine. The β-glucuronidase enzyme produced by intestinal bacteria cleaves the irinotecan metabolite SN-38G into a toxic form that damages the colonic mucosa and causes diarrhea, leading to a risk of subsequent bacterial translocation from the intestine and potential systemic infection [36]. According to the studies, antibiotics or modification of gut microbiome significantly alleviated the gastrointestinal toxicity in cancer patients [37]. Additionally, the use of indigestible fiber has proven to be beneficial, mainly due to the positive effect of short-chain fatty acids (SCFA) on intestinal mucosa. Several studies reported the reduced risk of developing irinotecan toxicity after changing eating habits, using appropriate probiotics and optimal butyrate intake [38,39].

Besides chemotherapeutic agents, drugs related to long-standing type 2 diabetes mellitus (DM), a chronic condition associated with an increased risk and worse PDAC outcomes of PDAC [40,41], suggest being beneficial in decreasing the incidence of PDAC. In particular, antidiabetic drug metformin (1,1-dimethylbiguanide hydrochloride) showed improved survival in locally advanced PDAC patients [42]. Accordingly, a propensity score analysis of 1916 PDAC patients with pre-existing DM treated with metformin and other DM medications (1098 and 818 patients, respectively) reported increased survival among the metformin-treated group [43]. Both preclinical and clinical studies suggest the association between gut microbial changes and the antidiabetic effect of metformin [44,45,46,47]. A comprehensive metagenomic and targeted metabolomic analysis of individuals with newly diagnosed type 2 DM showed that metformin interacted with different gut bacteria through the regulation of metal homeostasis, and significantly increased butyrate and propionate levels in patients. Moreover, fecal transfer of metformin-altered microbiota to germ-free mice led to an improvement in glucose tolerance [48]. Interestingly, Chang et al. demonstrated a chemopreventive effect of metformin on PDAC development in pancreatic cancer mouse model subjected to an obesogenic diet high in fat and calories (HFCD) [49]. In a recent study, HFCD-induced changes in diversity and composition of duodenal microbiome were prevented by metformin administration in a relevant mouse model, suggesting that the microbiome might play an important role in metformin-associated PDAC chemoprevention [50].

### 3.2. Microbiome and Immunotherapy

Breakthrough findings demonstrating the effect of microbiome on the efficacy of immunotherapy came from animal studies. Mice housed in germ-free conditions as well as animals treated with broad-spectrum antibiotics showed reduced effects of immunotherapy by a combination of TLR9 antagonist and anti-interleukin-10 antibody. The results suggest that intact intestinal microflora mediate the therapeutic effects of CpG-oligonucleotide immunotherapy through modulation of cellular functions in the tumor microenvironment [35]. Similarly, the ineffectiveness of cancer immunotherapy directed against the major negative regulator of T cell activation CTLA-4 was observed when applied to antibiotic-treated animals or germ-free mice. The presence of *Bacteroides* spp. has been shown to be an essential factor in the antitumor efficacy of CTLA-4 blockade, since fecal microbiota transplantation (FMT) of human *Bacteroides* spp.-rich feces significantly improved the outcome of transplanted animals [51]. These observations were supported by mouse models bearing melanoma, which demonstrated the role of *Bifidobacterium* spp. in enhancing antitumor immunity and the efficacy of PD-L1 blocking therapy [52]. Enhanced clearance of colon adenocarcinoma cells after immune checkpoint inhibitor (ICI) therapy has been achieved by the colonization of mice with IFNɤ^+^ CD8^+^ T-cell-inducing bacteria strains, isolated from healthy human microbiota as a consortium of 11 bacterial strains (four non-*Bacteroidales* and seven *Bacteroidales* spp.) [53]. Importantly, Pushalkar et al. reported an association between microbiome and immunotherapy in a mouse model of PDAC showing a synergistic effect of antibacterial treatment with αPD-1 therapy on tumor size among antibiotic-treated animals [54]. Recently, an association between the microbiome-derived inosine and enhanced efficacy of checkpoint blockade immunotherapy in mice with colorectal, bladder, and melanoma tumors has been discovered [55].

The results from animal models were firstly supported by metagenomic and metabolomic profiling in melanoma patients treated with a combination of anti-PD1 and anti-CTLA4 immunotherapy. According to the findings, the microbiome of ICI responders for all types of therapies was enriched for *Bacteroides caccae* [56]. Accordingly, further clinical studies comprising the patients with metastatic melanoma, nonsmall cell lung cancer, and renal cell carcinoma, reported the differences in the microbiome of patients responding to ICIs targeting PD-1/PD-L1 and of nonresponders [57,58]. Matson et al. showed that an abundance of *Bifidobacterium longum*, *Collinsella aerofaciens*, and *Enterococcus faecium* in commensal microbial composition was associated with an increased anti–PD-1 efficacy in metastatic melanoma patients. Moreover, FMT from responding patients to germ-free mice resulted in gut microbiome reconstitution followed by improved tumor control in mice after microbial transplantation [59].

## 4. Oral and Gut Microbiome in Pancreatic Cancer

**Figure 1:**
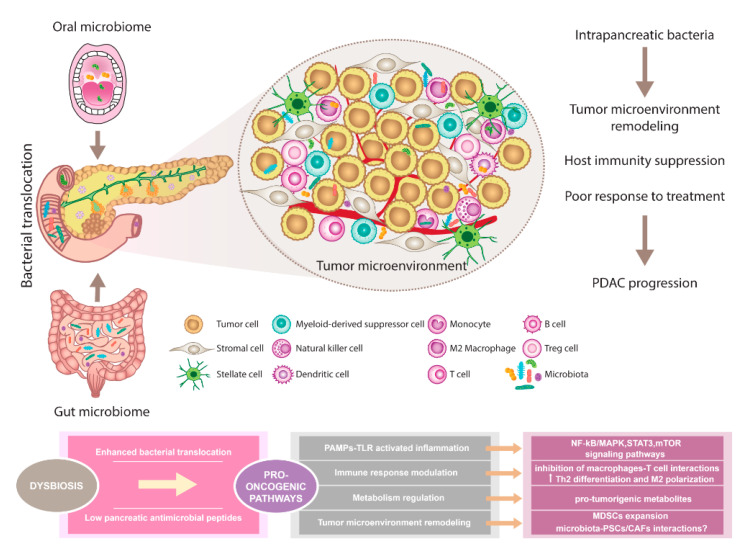
The pancreatic microbiota as a component of the tumor microenvironment. Comprehensive microbial studies support the existence of constant interactions between oral, gut, and pancreatic microbiome. Dysbiosis contributes to multiple changes associated with PDAC. Disruption of gut microbiota leads to enhanced bacterial translocation and subsequent activation of TLRs signaling pathway in the pancreatic environment. Besides, decreased SCFA metabolites negatively influence the production of pancreatic antimicrobial peptides. Microbiota-derived signals might affect pancreatic oncogenesis via NF-kB/MAPK, STAT3, or mTOR tumor-related inflammatory pathways. Moreover, immune response modulation leading to reduced Th1 CD4+ and CD8+ T cell differentiation and increased Th2 levels as well as the production of pro-tumorigenic metabolites might represent the pro-oncogenic mechanisms. Since microbiota has been recognized as an important part of the PDAC microenvironment, possible interactions with PSCs/CAFs need to be considered and further evaluated. Importantly, microbiota-dependent remodeling of tumor microenvironment towards PDAC immunosuppression has been reported, suggesting the complex interplay between all components within the tumor might affect the sensitivity to PDAC treatment. Abbreviations: CAFs, cancer-associated fibroblasts; MAPK, a mitogen-activated protein kinase; MDSCs, myeloid-derived suppressor cells; mTOR, the mammalian target of rapamycin; NF-kB, nuclear factor kappa-light-chain-enhancer of activated B cells; PAMPs, pathogen-associated molecular patterns; PDAC, pancreatic ductal adenocarcinoma; PSCs, pancreatic stellate cells; SCFA, short-chain fatty acid; STAT3, signal transducer and activator of transcription 3; Th2 cells, T helper 2 cells; T reg cells, regulatory T cells; TLRs, Toll-like receptors.

The composition of oral, gut, and pancreatic microbiome in PDAC patients showed diverse alterations compared to healthy individuals. Prior studies support the existence of complex interactions between various host microbiomes and the pancreas. This communication shapes immune regulation, influences the gastrointestinal microbiota via antimicrobial peptides derived from the pancreas, and modulates the effect of PDAC therapy (Figure 1).

Oral health status with regard to inflammation of the gingiva [60,61], periodontal disease [62,63], and tooth loss [64,65] represents independent risk factors for PDAC. The exact mechanisms by which oral microbiota reach the pancreas are still unknown, but the proposed mechanisms involve the translocation via biliary/pancreatic ducts or through the blood circulation [66]. Variations in salivary microbiota from patients with pancreatitis and PDAC support the possibility of using salivary microbial biomarkers for systemic diseases prediction [67]. Interestingly, the associations between oral pathogens *Porphyromonas gingivalis*, *Fusobacterium*, *Neisseria elongata*, and *Streptococcus mitis* and PDAC tumorigenesis have been evaluated. In this context, *P. gingivalis* showed a strong positive correlation with PDAC susceptibility. A detailed meta-analysis of 49 case-control studies comprising 5924 individuals (patients with periodontal disease and healthy controls) found a strong correlation between the presence of *P. gingivalis* and periodontal diseases [68]. Importantly, *P. gingivalis* was associated with an increased risk of PDAC with 59% greater risk of PDAC in a cohort of 361 PDAC patients compared to 371 matched-healthy controls [69]. Moreover, *Fusobacterium* spp. have been detected in pancreatic cancer tissue and show significant association with a worse prognosis [70].

Data from murine models demonstrated that the gut microbiome can colonize pancreatic tumors and modify its overall intratumoral bacterial composition. *Helicobacter pylori* became an established risk factor for gastric cancer and its role in PDAC development has also been assessed. One of the first observations came from analysis of IgG antibodies against *H. pylori* by enzyme-linked immunosorbent assays. High seropositivity was detected in blood samples from patients with pancreatic and gastric cancer (65% and 69%, respectively) compared to other individuals [71]. A prospective study on more than 50,000 male participants revealed an increased risk of pancreatic tumors related to gastric ulcer. As authors acknowledged, the elevated risk might be associated with greater endogenous nitrosamine formation and inflammatory response due to *H. pylori* infection [72]. Some studies and meta-analyses confirmed the link between *H. pylori* and increased risk of PDAC [73,74,75], while others did not support these findings [76]. Very recently, Hirabayashi et al. performed a large population-based cohort study in a Japanese population showing *H. pylori* infection and atrophic gastritis (AG) were not generally associated with the risk of pancreatic cancer; however, significant positive correlation was detected among current smokers with AG [77]. Due to the conflicting results, the further research is warranted to determine the true impact of *H. pylori* in pancreatic carcinogenesis.

## 5. Pancreatic Tumor Micro- and Mycobiome

Reciprocal interactions between the gut microbiome and immune system represent the key mechanism by which human microbiota influence the outcome of cancer patients. However, the impact of bacteria located in pancreatic cyst fluids, pancreatic neoplasms, or within the tumors is less known. The microbiome analysis of patients undergoing pancreaticoduodenectomy revealed distinct bacterial populations in fluids collected from the bile duct, pancreas, and jejunum [78]. Gaiser et al. observed significantly higher intracystic bacterial 16S DNA copy number and IL-1β quantification in intraductal papillary mucinous neoplasms (IPMN) with high-grade dysplasia and IPMN with cancer compared to non-IPMN neoplasms. Additionally, co-occurrence and enrichment of oral bacterial taxa including *Fusobacterium nucleatum* and *Granulicatella adiacens* was found in cyst fluid from IPMN with high-grade dysplasia [79]. Detection of bacterial 16S ribosomal DNA by qPCR in tumor samples from 113 PDAC patients revealed bacterial DNA in 86/113 (76%) PDAC samples compared to 3/20 (15%) in normal pancreas controls obtained from organ donors (*p* < 0.005). Fluorescence in situ hybridization (FISH) with fluorescent 16S rRNA-targeted probes, and immunohistochemistry, using an antibacterial lipopolysaccharide (LPS) antibody confirmed the intratumoral localization of bacteria in a PDAC cohort [80]. 

The study by Thomas et al. detected the pancreatic microbiota in human specimens pathologically confirmed as normal tissue, pancreatitis, or PDAC in a small cohort after surgical resection. However, 16S rRNA gene sequencing did not find a difference between the microbial composition in PDAC and non-PDAC tissue [81]. Recently, Nejman et al. performed the most comprehensive analysis of the tumor microbiome to date, showing a distinct composition of intratumoral microbiota relating to cancer type. Interestingly, intracellular bacterial localization in both cancer and immune cells was confirmed by visualization methods and culturomics. The microbial analysis of 1526 tumor samples and adjacent normal tissue from breast, lung, ovary, pancreas, melanoma, bone, and brain cancer patients revealed that breast, pancreatic, and bone tumors had the highest proportion of tumors positive for bacterial DNA assessed by 16S rDNA qPCR method. As shown, the microbiome from pancreatic carcinomas had copied the main signature of the normal duodenal microbial composition with dominance of Proteobacteria phylum. Moreover, breast and pancreatic tumor samples were found to be enriched by *Fusobacterium nucleatum* [82]. 

A comparison of fecal microbiota from PDAC patients and healthy controls showed a higher abundance of Proteobacteria, Actinobacteria, Fusobacteria, and Verrucomicrobia in the gut of cancer patients. Interestingly, Proteobacteria (45%), Bacteroidetes (31%), and Firmicutes (22%) were most abundant within intratumoral microbiome of PDAC patients [54]. This finding confirmed the previous results by Geller et al. considering the possible mechanism of interaction between the two compartments by a retrograde bacterial translocation from the duodenum via pancreatic ducts [80]. 

Metagenomic analysis of 68 tumor samples from PDAC patients showed the correlation between pancreatic tumor microbiome and patients’ survival. Microbial comparison of two cohorts identified as short-term survivors (STS) and long-term survivors (LTS) showed that tumors of LTS were characterized by a significantly higher alpha diversity (*p* < 0.05 for each alpha diversity index), and infiltration with cytotoxic CDþ/killer T cells compared to STS. Importantly, both cohorts were age, gender, stage, and treatment matched. According to the findings, tumor bacterial diversity was associated with OS (median survivals of the patients with high versus low alpha diversity were 9.66 and 1.66 years, respectively). This finding correlates with results showing that low bacterial diversity is linked to a worse outcome for cancer patients, especially those with hematologic malignancies. The ability of gut microbiome to modulate the pancreatic tumor microbiome and influence the tumor growth was confirmed by animal models with FMT from pancreatic STS, LTS, and healthy donors. Moreover, the positive effect of FMT on intratumoral immune cell infiltration has been observed [83].

However, not only gut microbiome but also gut mycobiome can directly influence the pancreatic microenvironment. Oral gavage of control and pancreatic-tumor bearing mice with GFP-labeled *Saccharomyces cerevisiae* indicated that endoluminal fungi could access the pancreas. According to the findings, both mouse and human PDAC tumors exhibited a greater than 3000-fold increase in abundance of fungi compared with normal pancreas. Furthermore, a distinct composition of mycobiome and enrichment with *Malassezia* spp. has been observed in adenocarcinoma samples. The ablation experiments confirmed the accelerated oncogenesis after *Malassezia globosa* repopulation in murine models, suggesting the participation of the C3 complement cascade via mannose-binding lectin activation in promoting PDAC [84].

## 6. Novel Treatment Approaches for Pancreatic Ductal Adenocarcinoma

Most chemotherapeutics and immunotherapeutics efficient in other malignancies display limited efficacy in PDAC, even in patients after R0 resection with pathologically tumor-free surgical margins [85,86]. Since, dense desmoplastic stroma represents one of the main factors responsible for the failure of currently applied PDAC treatment, focusing on nonmalignant cells within the tumor microenvironment might represent an option to overcome PC chemoresistance and immune tolerance. In this context, pancreatic stellate cells (PSCs), as the main source of the stromal collagen, play a key role in the PDAC microenvironment [87]. Novel therapeutic approaches such as stroma-targeting therapy, immunotherapy, and neoantigen vaccines need to be able to face the highly immunosuppressive PDAC tumor microenvironment. These studies recognize the microbiota as an important component of the PDAC microenvironment, and its role in the activation of PSCs needs to be evaluated. Moreover, the microbial remodeling of the tumor microenvironment towards immune tolerance might be associated with the inefficiency of antitumor immunotherapy [88]. Thus, a microbiota-derived approach should also be taken into account and numerous clinical trials evaluating the effect of microbiome in pancreatic cancer are currently ongoing (Table 1).

### 6.1. Tumor Microenvironment-Driven Therapy Response

The main drivers of pancreatic homeostasis and regeneration are plasticity and heterogeneity of acinar cells, as, in contrast to other organs of the gastrointestinal tract, the pancreas lacks a defined stem cell compartment [89]. Activated by external and internal stimuli such as inflammation or damage, acinar cells transdifferentiate to more epithelial (ductal-like) phenotypes in a process called acinar-to-ductal metaplasia [90]. During this process, acinar cells acquire “progenitor cell-like” characteristics, which make them more vulnerable to pro-oncogenic hits, including activating mutations or epigenetic changes. The main feature of PDAC tumors is an extensive desmoplastic stroma, constituting up to 90% of tumor volume. It is considered to originate from distinct subtypes of cancer-associated fibroblasts (CAFs) with either myofibroblastic or inflammatory phenotypes (myoCAFs or iCAFs, respectively) [91]. The main source of CAFs appears to be the pancreatic stellate cells, which, when activated, start depositing huge amounts of extracellular matrix composed of collagens, laminins, fibronectins, or hyaluronan [92,93].

Microbiota participates in processes related to tumor progression, and the possible role of microbial metabolites in shaping the tumor microenvironment has been already reviewed [94]. Interestingly, deoxycholic acid (DCA), a gut microbiota metabolite derived from the metabolism of unabsorbed primary bile acids, increases the invasiveness and proliferation of colorectal cancer cells via transcription activation of cyclooxygenase 2 (COX-2) in CAFs [95]. In addition, COX-2 activation lead to increased production of prostaglandin 2 a key mediator in fibrotic processes in fibrosis and cancer [96]. Zambirinis et al. showed a broad expression of TLR9 early during pancreatic transformation. Moreover, TLR9 ligation activated PSCs to became fibrogenic and secrete chemokines promoting epithelial cell proliferation. Protumorigenic effects of TLR9-activated PSCs on the epithelial compartment were found to be mediated via C-C Motif Chemokine Ligand 11 (CCL-11) [97]. Interestingly, previous results showed that gut or systemic microbiota might be a potential source of TLR9 ligands in invasive or preinvasive PDAC [98]. Thus, a deeper understanding of intratumoral cross-talk between PSCs or CAF and microbiome is challenging. Since dense desmoplasia severely limits the efficacy of PDAC treatment and CAFs suggest to be involved also in cancer spread [99], further studies of all possible factors contributing to CAFs activations are highlighted.

Hypoxia is one of the key features of the PDAC microenvironment, originating from desmoplasia-associated hypervascularization and favoring desmoplastic progression by pancreatic stellate cells activation [100]. Hypoxia and desmoplasia are accompanied by a strong accumulation of myeloid cells. Macrophages recruited to the tumor microenvironment adopt an immunosuppressive, proangiogenic state, and block entry of CD4+ T cells into the microenvironment, thus supporting PDAC progression [101]. Systemic frequencies of granulocytes and monocytes are elevated in patients, with pathologic activation and immunosuppressive function, classified as polymorphonuclear myeloid-derived suppressor cells (MDCSs) [102]. Moreover, the PDAC microenvironment represents a comparable degree of cytokine complexity, with dominating cytokines such as TGF-β, IL-6, IL-8, IL-10, IL-35, granulocyte-macrophage colony-stimulating factor (GM-CSF), CXC-chemokine ligand 1 (CXCL-1), CC-chemokine ligand 2 (CCL-2), and CXCL-13 (chemokine (C-X-C motif) ligand-13) [103]. These orchestrate the recruitment and education of innate and adaptive immune cells and their cross talk with CAFs and other cells in the tumor microenvironment.

Impaired drug delivery has been repeatedly associated with a dense desmoplastic reaction in PDAC and high interstitial pressure, the role of matrix components and presence of cancer stem cells (CSCs) that increase drug resistance [104]. On the other hand, high stromal content also correlated with favorable outcomes in resected patients, with less abundant stromal content in metastatic PDAC [105]. These findings suggest divergent roles of the heterogeneous CAF populations, perhaps including subtypes that support and others that suppress tumor growth [106].

### 6.2. Microbiota-Dependent Efficacy of PDAC Treatment

Oral gavage with fluorescently-labeled *Enterococcus faecalis* or *Escherichia coli* confirmed that gut bacteria were able to access the pancreas and might affect the pancreatic microenvironment. Amplicon 16S rRNA gene sequencing showed distinct stage-specific gut and pancreatic microbiome in tumor samples, inducing intratumoral immune suppression and PDAC progression. Furthermore, fecal transfer from PDAC-bearing mice contributed to disease progression. According to the results, deficiency in pattern recognition receptor (PRR) signaling slowed PDAC progression [97,107]. Activation of PRRs and TLR ligation by microbial lipopolysaccharides and flagellins in peritumoral milieu might promote tumor microenvironment reprogramming, and accelerate the PDAC tumorigenesis [54]. Moreover, modification of microbiome by oral antibiotic administration induced tumor microenvironment remodeling leading to a reduction in MDSC and an increase in M1 macrophage differentiation and intratumoral CD4+ and CD8+ T cell activation. Together, bacterial ablation enhanced antitumor immunity and increased susceptibility to αPD-1 immunotherapy by upregulating PD-1 expression on effector T cells in a PDAC orthotopic mouse model. Hence, a combination of specific microbiota ablation with checkpoint-directed immunotherapy might represent a potential treatment strategy for PDAC patients [54].

Gemcitabine is used as a key chemotherapeutic agent in the treatment of PDAC patients, therefore a deeper understanding of the mechanism of resistance would be of particular interest. Expression of the bacterial cytidine deaminase (CDD_L_) by intratumor Gammaproteobacteria was shown to be responsible for gemcitabine resistance in a mouse model of colorectal cancer due to the ability to metabolize the chemotherapeutic drug gemcitabine (2′,2′-difluorodeoxycytidine) into its inactive form (2′,2′-difluorodeoxyuridine). Moreover, cotreatment with ciprofloxacin has been shown to abrogate gemcitabine resistance. Interestingly, 16S rRNA sequencing of 65 human PDAC tumors identified Gammaproteobacteria (mostly members of Enterobacteriaceae and Pseudomonadaceae), as the most common bacterial taxa representing 51.7% of all reads. Geller et al. suggested the potential role of intrapancreatic microbiota in modulation of tumor resistance to gemcitabine, since cultivation of bacteria from 15 fresh PDAC tumors with colorectal carcinoma cell cultures mediated a complete resistance to a chemotherapeutic agent [80]. Discoveries from PDAC-bearing mice on gemcitabine-treated and nontreated controls revealed the substantial modifications in the gut bacterial composition. The shift towards an inflammation-related bacterial profile with increased Proteobacteria and Verrucomicrobia phylum is assumed to aggravate the pancreatic inflammatory state. Furthermore, bacterial translocation through the bloodstream or direct reflux through the pancreatic ducts might promote immune remodeling of the peritumoral microenvironment [108].

## 7. Gut Microbiota Modulation by Antibiotics, Probiotics, and Fecal Transplantation

**Figure 2:**
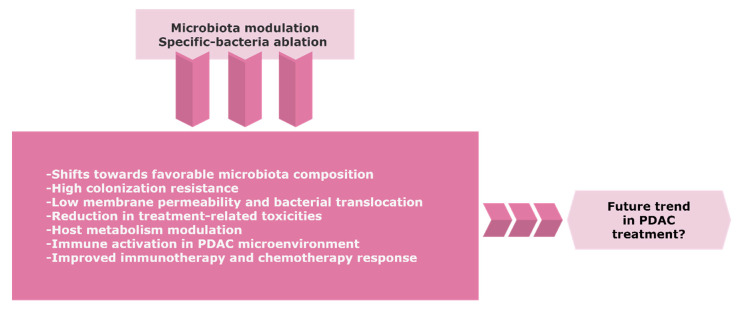
The possible trend of the gut and/or tumor microbiome modulation in PDAC. Precise targeting of microbiota composition might represent a novel approach to improve the therapeutic efficacy and clinical outcome for PDAC patients. Further research and randomized control trials with careful benefit-risk assessment are warranted due to the considerable risks of infection in immunosuppressive cancer patients. Abbreviations: PDAC, pancreatic ductal adenocarcinoma.

Re-establishing an effective intestinal ecosystem with a favorable enteric microbiota might increase the efficacy of cancer treatment (Figure 2). Despite the emerging role of the microbiome in PDAC, there is a limited number of controlled trials with a consistent design regarding the potential role of the gut and/or tumor microbiome modulation towards tumor progression or improving the sensitivity to therapeutics.

Oral antibiotics lead to an antitumor immune activation and restrained tumor burden in mice models bearing PDAC [109]. Coadministration of the PDAC drug gemcitabine with ciprofloxacin significantly reduced the level of detectable bacteria via in vivo imaging, and improved the response to the chemotherapeutic agent in colon mouse models [80]. In addition, bacterial ablation via oral antibiotics was found to be protective in pancreatic tumorigenesis and to augment the sensitivity to immunotherapy [54]. Mohindroo et al. retrospectively analyzed the clinical data of 148 metastatic PDAC patients (135 patients exposed to antibiotics) showing prolonged OS and PFS (progression-free survival) after macrolide consumption longer than 3 days [110]. However, a retrospective single-center cohort study on resectable PDAC patients found that tetracycline treatment was associated with clinically significant decreased PFS and statistically significant worse OS [111]. Recently, the reanalysis of the comparator arm of the MPACT clinical trial (comprising 430 metastatic PDAC patients on antibiotic therapy) demonstrated increased gemcitabine-associated toxicity during and after antibiotic exposure [112].

Numerous studies highlight the positive effects of probiotics and prebiotics on gastrointestinal cancers through the activation of the host’s immune system, maintenance of intestinal barrier integrity, reduction in microbial activity by decreased intestinal pH, as well as inhibition of bacteria involved in the conversion of procarcinogens to carcinogens [113]. Probiotics are described as “mono- or mixed cultures of live microorganisms able to beneficially affect the host by improving the properties of the indigenous flora” [114]. Decreasing the treatment-associated gastrointestinal toxicity is considered to be the main reason for probiotic administration in cancer patients. According to a single-center survey study with 499 participants suffering from distinct malignities, probiotic supplementation was utilized by 28.5% of all respondents [115].

Bacterial translocation is thought to be a possible route of communication between the gut and pancreatic microbiota. Hence, the effects of probiotic modulation in patients with pancreatitis have been evaluated as a risk factor for PDAC development. The first randomized, controlled, and double-blind study in a small cohort of 45 patients with severe acute pancreatitis (SAP) reported a significant reduction in pancreatic sepsis and the number of surgical interventions [116]. However, these results were not able to be reproduced in a second trial [117]. Importantly, the multicenter, randomized, and double-blind versus placebo PROPATRIA study, comprising 296 patients, reported that probiotic prophylaxis did not reduce the risk of infectious complications and was associated with an increased risk of mortality in patients with predicted SAP [118]. Moreover, the result of meta-analysis of six clinical trials found no significant effects of probiotics on the clinical outcomes of patients with SAP [119].

Fecal microbiota transplantation (FMT) contains a greater quantity of microbiota than commonly used probiotic supplements and may represent a promising trend in overcoming the immunosuppression and resistance to therapy in cancer patients likely to have relatively short survival [120]. Animal studies suggest the protective effect of gut and tumor bacteria in PDAC patients who had survived more than 5 years without evidence of disease (long-term survivors). Mice that received FMT from patients with advanced disease harbored much larger tumors compared to the animals receiving FMT from long-term survivors of PDAC or healthy controls [83]. To evaluate the results from preclinical findings, the first clinical trial on resectable PDAC patients receiving FMT from healthy donors delivered through both colonoscopy and oral pills is in preparation.

## 8. Conclusions and Future Perspectives

The application of individual microbial signatures as the early biomarkers for cancer development and prognostic indicators of treatment response is still an important challenge for microbiota research. Thus, longitudinal epidemiological studies concerning the significant rearrangements in the microbial composition are highly warranted. An association between organ-specific oncogenesis and particular microbial taxa might represent the key to the understanding of underlying mechanisms. Especially in cancers with poor prognosis such as PDCA, where the risk factors are far less known, the elucidation of the role of microbiota not only in initiation but also in disease progression would be of the highest interest.

Due to the contribution of microbiota to an enormous variety of metabolic and immunological pathways, the particular composition of patient´s microbiome should be taken into account to achieve the most efficient therapy response. Accordingly, some studies have already suggested the prognostic value of pancreatic tumor microbiome for cancer treatment efficacy. Pilot results showed that bacteria found in PDAC tumor samples could modulate sensitivity to therapeutic agents. The existence of communication between the gut and organ-unique microbiome brings new opportunities for cancer research, and targeting the commensal microbiota represents a big challenge for precision medicine in the near future. Hence, a combination of chemotherapy and immunotherapy with proper microbiota modulation might improve the efficacy of cancer treatment and outcome for PDAC patients.

## Figures and Tables

**Table 1:** Pancreatic cancer, solid tumors, and the microbiome. The table summarizes the list of ongoing and completed clinical trials dealing with the impact of microbiome on the risk, prognosis, and treatment efficacy in pancreatic cancer and solid tumors (according to http://clinicaltrials.gov/).

Study	Study Design	Disease	Purpose	Patients (n)	Intervention	Study Status
**NCT03302637**	A prospective, observational, case-control study	Pancreatic cancer	To determine the relationship of oral and pancreatic microbiome, and their impact on pancreatic cancer risk.	732	16S rRNA gene sequencing assay, extraction of genomic DNA from oral samples	Completed-results not posted
**NCT04274972**	A prospective, observational, cohort study	Pancreatic cancer	Qualitative and quantitative analysis of the pancreatic microbiome in patients with PDAC submitted to pancreaticoduodenectomy, sampling the lesion intraoperatively.	Estimated enrollment 20	The oral and rectal microbiome samples will be collected preoperatively. The PDAC tissue from the surgical specimen, the intestinal mucosal tissue from the enteric side of the pancreatic anastomosis, and the bile sample will be collected intraoperatively. On the 30th postoperative day, the oral and rectal samples will be repeated.	Recruiting
**NCT04189393**	A prospective, observational, cohort study	Gastrointestinal cancer	To assess changes in microbiome composition during surgical treatment quantified as alpha diversity by 16S rRNA sequencing.	Estimated enrollment 60	Four types of samples will be collected for microbiome analysis: saliva, feces, intraoperative mucosal swabs, and drain fluid	Active, not recruiting
**NCT04193904**	An interventional open-label phase I study	Pancreatic cancer	To assess the safety of MRx0518 in combination with hypofractionated preoperative radiation through the collection of adverse events.	Estimated enrollment 15	Drug: MRx0518 Radiation: hypofractionated preoperative radiation	Recruiting
**NCT04579978**	A prospective, observational, cohort study	Advanced Solid tumors	To investigate relative abundance and composition of immunotherapy response-associated bacterial species in patients with advanced/unresectable or metastatic solid tumors.	Estimated enrollment 60	Fecal microbial composition analyzed by 16S rRNA and metagenomic sequencing.	Recruiting
**NCT04243720**	A prospective, observational, cohort study	Solid tumors	To assess several outcomes; fecal microbiome changes associated with primary or acquired resistance to immunotherapy given alone or in combination in patients with advanced solid tumors.	Estimated enrollment 100	Stool sample will be collected for DNA extraction.	Recruiting
**NCT01706393**	An interventional, randomized study	Solid tumors	To evaluate the effect of probiotics to change the intestinal microbiome in patients undergoing concurrent pelvic/abdominal RT.	Estimated enrollment 26	Dietary supplement: probiotics (six probiotic cultures); 2 capsules bid orally for 6 weeks, 1 capsule (500 mg). The subjects will start eating probiotics 1 week prior of radiation therapy.	Unknown
**NCT04600154**	An interventional, randomized study	Pancreatic cancer	To evaluate the effects of MS-20 on gut microbiota and risk/severity of cachexia in patients receiving chemotherapy for pancreatic cancer.	Estimated enrollment 40	MS-20 or placebo will be orally administered twice per day in treatment period.	Active, not recruiting
**NCT03840460**	A prospective observational cohort study	Pancreatic cancer	To describe the incidence and distribution of biomarkers and identify molecular subtypes in a large, multicenter population of patients with pancreatic cancer or precursor lesions. To identify the molecular predictors of response or toxicity to standard of care anticancer therapies in PDAC/PanNET.	Estimated enrollment 200	Blood, urine, stool, saliva, bile, and tissue samples from patients undergoing a tissue biopsy or surgery for suspected or known pancreatic cancer will be collected. Molecular analyses including miRNA analysis, DNA and RNA sequencing, nanostring, RT-PCR, and immunohistochemistry will be carried out.	Recruiting
**NCT03891979**	A pilot study	Pancreatic cancer	To determine the change in immune activation in pancreatic tumor tissue following treatment with antibiotics and pembrolizumab.	0	Drug: pembrolizumab Drug: ciprofloxacin 500 mg PO BID days 1–29 Drug: metronidazole 500 mg PO TID days 1–29	Withdrawn (Suspended due to Primary Investigator’s decision)
**NCT04203459**	A prospective observational cohort study	Pancreatic cancer	To study the mechanism of enhancing the antitumor effects of human chimeric antigen receptor T cells on pancreatic cancer by gut microbiota regulation.	Estimated enrollment 80	The collected blood and tissue will undergo molecular analyses, including but not limited to, miRNA analysis, DNA and RNA sequencing, nanostring, real-time PCR, and immunohistochemistry.	Recruiting
**NCT01562626**	An interventional phase I/II study	Solid tumors	To evaluate the safety and tolerability of APS001F treatment plus 5-FC and maltose.	Estimated enrollment 75	Drug: APS001F APS001F infusion on days 1, 2, and 3 of each 28-day cycle Drug: 5-FC oral doses on days 11–15 and 18–22, each 28-day cycle Drug: 10% maltose 10% maltose infusion will be administered on days 1–5, 8–12, and 15–19, each 28-day cycle	Recruiting
**NCT03637803**	An interventional phase I/II study	Solid tumors	To assess the safety and tolerability of MRx0518 in combination with pembrolizumab through the collection of adverse events.	Estimated enrollment 132	Drug: MRx0518 Drug: pembrolizumab 25 mg/1 mL intravenous solution	Recruiting

Abbreviations: PDAC, pancreatic ductal adenocarcinoma; rRNA, ribosomal ribonucleic acid; RT, radiation therapy; 5-FC, 5-fluorocytosine; RT-PCR, real-time polymerase chain reaction; miRNA, microRNA; DNA, deoxyribonucleic acid.
